# Temporal Predictive Codes for Spoken Words in Auditory Cortex

**DOI:** 10.1016/j.cub.2012.02.015

**Published:** 2012-04-10

**Authors:** Pierre Gagnepain, Richard N. Henson, Matthew H. Davis

**Affiliations:** 1 MRC Cognition and Brain Sciences Unit, Cambridge CB2 7EF, UK

## Abstract

Humans can recognize spoken words with unmatched speed and accuracy. Hearing the initial portion of a word such as “formu…” is sufficient for the brain to identify “formula” from the thousands of other words that partially match [1–6]. Two alternative computational accounts propose that partially matching words (1) inhibit each other until a single word is selected (“formula” inhibits “formal” by lexical competition [7–9]) or (2) are used to predict upcoming speech sounds more accurately (segment prediction error is minimal after sequences like “formu…” [10–12]). To distinguish these theories we taught participants novel words (e.g., “formubo”) that sound like existing words (“formula”) on two successive days [13–16]. Computational simulations show that knowing “formubo” increases lexical competition when hearing “formu…”, but reduces segment prediction error. Conversely, when the sounds in “formula” and “formubo” diverge, the reverse is observed. The time course of magnetoencephalographic brain responses in the superior temporal gyrus (STG) is uniquely consistent with a segment prediction account. We propose a predictive coding model of spoken word recognition in which STG neurons represent the difference between predicted and heard speech sounds. This prediction error signal explains the efficiency of human word recognition and simulates neural responses in auditory regions.

## Highlights

► Lexical competition and segment prediction accounts of spoken word recognition ► Overnight consolidation of pseudowords modifies segment prediction ► Predictive coding simulates MEG data in superior temporal gyrus ► Prediction error supports word recognition without competition at the lexical level

## Results

### Computational Simulations of Spoken Word Recognition

**Figure 1:**
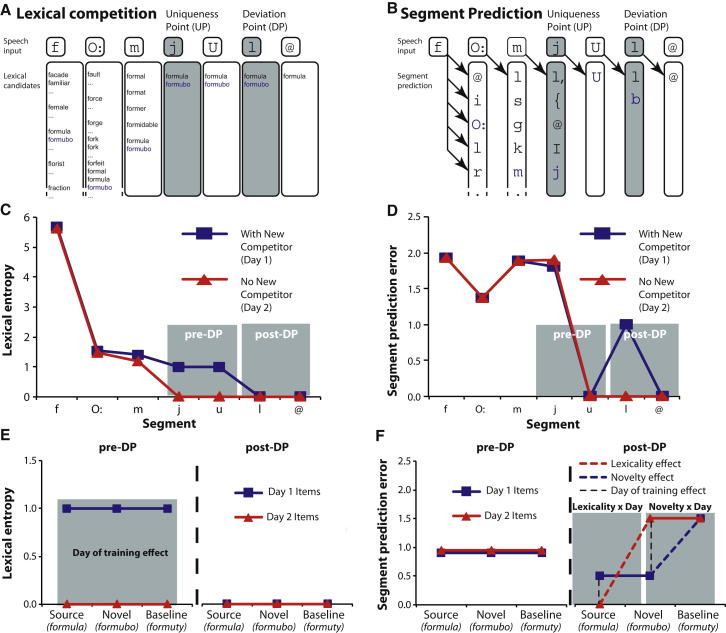
Computational Simulation of Lexical Competition and Segment Prediction The impact of an additional lexical candidate (formubo, in blue) is shown for (A) lexical hypotheses and (B) segment predictions during recognition of the familiar word “formula” /fO:mjul@/. The addition of this novel word to the lexicon modifies the time course of neural responses to this familiar word as simulated by (C) lexical competition and (D) segment prediction accounts of word recognition. Computational simulations show differential timing and direction of modifications to lexical entropy and prediction error measures from these two accounts. The bottom panel shows experimental predictions for neural correlates of (E) lexical entropy and (F) segment prediction error measures for six critical conditions in our experiment, (source/novel/baseline items, trained on day1/day2; see Figure 2A) averaged over speech segments before and after the DP for the item set “formula,” “formubo,” and “formuty.” These profiles are typical of the pattern observed for all 216 triples in our item set. A neural implementation of the segment prediction account can be found in Figure 4 and a simulation for all items in Figure S3.

All current accounts of spoken word recognition propose that identification occurs once speech segments that uniquely identify a single item are heard (i.e., recognition occurs after the lexical uniqueness point [UP]; Figure 1A) [7–9]. However, the computational mechanism by which such “ideal observer” behavior is achieved differs between accounts. The dominant proposal in current computational models of spoken word recognition is that multiple lexical candidates are activated in parallel and compete through inhibitory connections [7] or other, functionally equivalent lexical mechanisms [8, 9]. These accounts therefore make the neural prediction that responses in anterior and inferior temporal regions contributing to lexical identification [17] or, in frontal regions involved in lexical selection [6], should be correlated with the degree of lexical uncertainty as quantified by lexical entropy (Figure 1C). An alternative account, however, proposes that prediction of upcoming speech segments is a key computation during lexical processing (Figure 1B) [10–12]. Lexical candidates that match the current speech signal generate predictions for upcoming segments that are confirmed or disconfirmed by subsequent input. By this account, lexically unique sequences allow for more accurate prediction of upcoming speech and lead to a reduction in “prediction error”—i.e., the difference between predicted and elicited pattern of activity by speech input—in regions involved in speech segment perception such as the STG [18–20].

**Figure 2:**
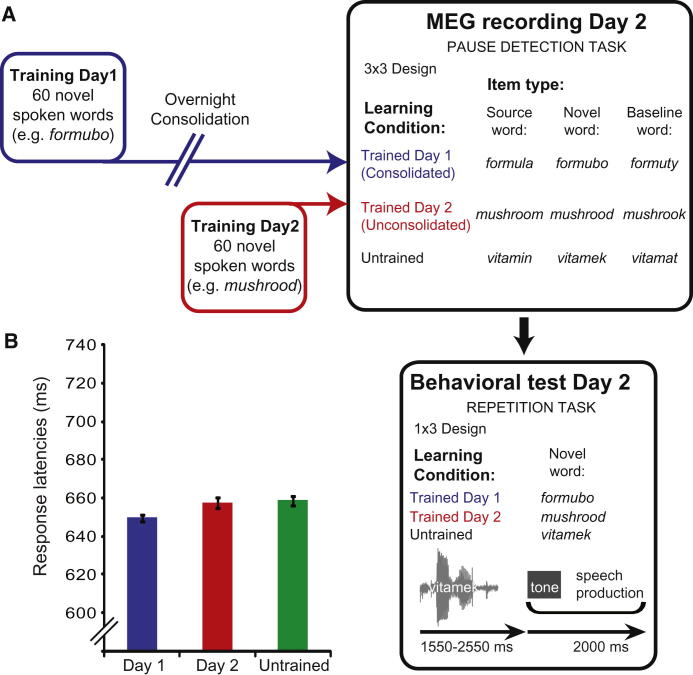
Experimental Design and Behavioral Results (A) Experimental design. Participants were trained during a phoneme-monitoring task on two distinct sets of novel spoken words on two successive days. MEG recording after the second training session consisted of a pause-detection task [21] with 17% target items not included in the analyses. Following MEG recording, participants performed a delayed repetition task on trained and untrained novel spoken words (see Supplemental Experimental Procedures, Section B.4, for further details). (B) Response latencies during delayed repetition for novel spoken words trained on day 1, day 2, or untrained. Error bars show ± within-participant SE.

To separate these two types of computation, we experimentally modified the natural organization of the lexicon by teaching participants two sets of fictitious novel words on successive days (e.g., “formubo” and “mushrood”) that closely overlap with one existing word (“formula” and “mushroom”) (Figure 2A). Once a word like formubo has been added to the lexicon (if learning is followed by overnight consolidation) [13–16], it will change the UP of formula to a later point at which new and existing words diverge (deviation point [DP]; see Figures 1A and 1B). Computational simulations (see below) show that learning and consolidation of a new item produces a transient increase in lexical entropy prior to the DP (because there are now two matching lexical candidates rather than one as previously). Lexical entropy is zero post-DP, when items can be uniquely identified (Figures 1C and 1E). Conversely, pre-DP prediction error is slightly decreased by the addition of a new candidate because post-UP segments can now be more confidently predicted (Figure 1D). However, a marked increase in prediction error is seen post-DP (Figures 1D and 1F), because there are two potential post-DP consonants (/l/ or /b/ for “formula” and “formubo”). Thus, although both increased lexical competition and prediction error may account for delayed behavioral responses to words that match many potential competitors [13–16], it is only by assessing neural responses time-locked before and after DP that these computations can be distinguished.

Pre- and post-DP magnetoencephalographic (MEG) responses to consolidated (day 1) novel words (“formubo”), source words (“formula”), and baseline nonwords (“formuty”) were therefore recorded during a pause-detection task [13, 21] and compared to equivalent items that were trained but remained unconsolidated (day 2) and items that had not been presented prior to the MEG (untrained; see Figure 2A and Supplemental Experimental Procedures available online). Not only do the lexical entropy and segment prediction accounts predict opposite effects of training (day 1 versus day 2) pre-DP, as explained above, but post-DP, the segment prediction account (but not lexical entropy account; Figure 1E) predicts a specific pattern comprising two interactions: (1) a cross-over “lexicality-by-day” interaction such that the difference between source versus novel items changes with day of training, and (2) a “novelty-by-day” interaction between novel versus baseline items and day of training (Figure 1F). The former interaction is driven by greater prediction error for (1) novel versus source unconsolidated (day 2) items (a “lexicality” effect), (2) day 2 versus day 1 novel items (due to stronger segment predictions for day 1 items), and (3) day 1 versus day 2 source items (due to the presence of two predicted segments for day 1 items), whereas the latter interaction is driven by greater error for baseline versus novel day 1 items (a “novelty” effect) (see Supplemental Experimental Procedures, Section B.1, for further explanation). MEG data was recorded from 24 right-handed participants in a study approved by the Cambridge Psychology Research Ethics Committee.

### Behavioral Effects of Consolidation

Behavioral evidence of overnight consolidation of newly learned words was assessed during a subsequent delayed repetition task (Figure 2A) [13]. One-tailed paired t tests on vocal response latencies (Figure 2B) showed significantly faster latencies for day 1 (M = 649 ms, SD = 174) relative to day 2 novel words (M = 657 ms, SD = 178), (t[18] = −1.87, p < 0.05), and relative to untrained novel words (M = 658 ms, SD = 176) (t[18] = −2.99, p < 0.005). No reliable difference was observed between day 2 and untrained novel words (t[18] = −0.23). These results replicate earlier findings; changes in word repetition latency [13] and cued word retrieval [16] parallel the overnight emergence of lexical integration effects (lexicalization) [14].

### Effects of Training Day on Pre-DP Neural Responses

We first computed the main effect of day 1 versus day 2 training on pre-DP neural responses, averaging responses over all three item types (source, novel, baseline) because these are lexically indistinguishable using pre-DP speech segments (given that the phoneticians who marked DP took account of coarticulation [22]; Supplemental Experimental Procedures, Section B.4.2). The global field power (GFP) (root-mean-square [rms] of data across all MEG gradiometers) showed no reliable differences between trained day 1 versus trained day 2 conditions when averaged over the pre-DP time window of interest for Z = −1.37, p = 0.17 (magnetometer data are reported in Supplemental Results, Section A.4.1). In fact, if anything, there was a numerical trend in the opposite direction to that predicted by lexical entropy accounts, i.e., greater GFP for trained day 2 versus trained day 1 (and versus untrained) conditions. Results of sensor-time analyses of rms-gradiometers also failed to show reliable difference (p corrected = 0.13).

**Figure 3:**
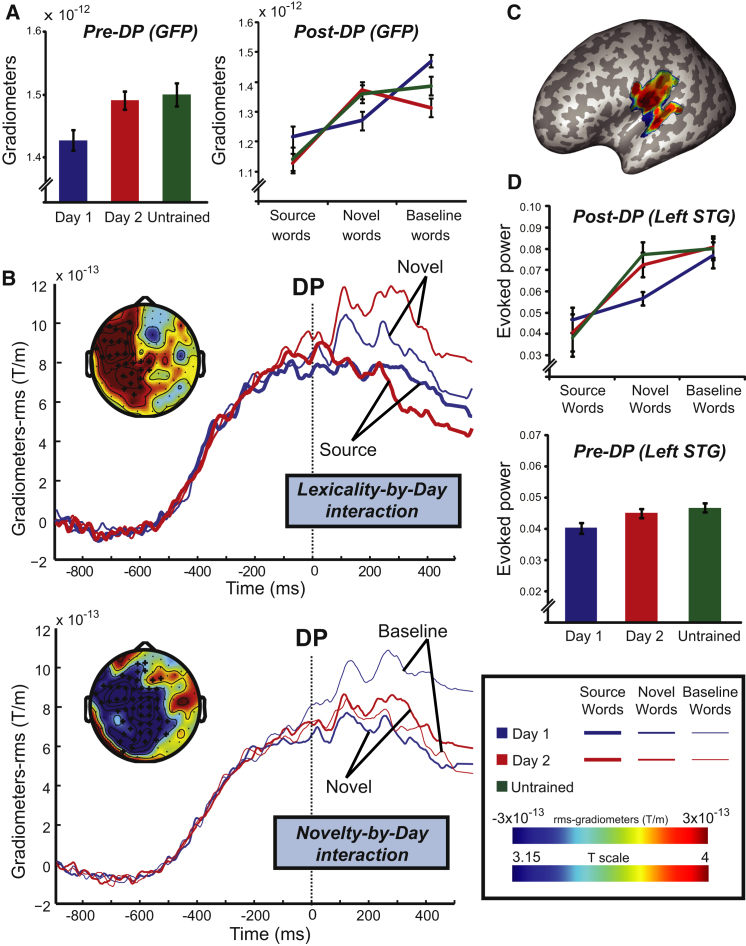
Pre- and Post-DP Neural Responses and Source Reconstruction (A) GFP of the gradiometers averaged over the pre-DP (100–200 ms post-UP locked epochs) and post-DP (100–500 ms) time windows of interest. These data are uniquely consistent with computations of prediction error derived from a segment prediction account (cf. Figures 1E and 1F). (B) Time-course plots of significant rms-gradiometer sensors (marked by a cross in the scalp topography) for the lexicality-by-day and novelty-by-day interactions. The x axis measures time relative to acoustic DP as marked by a trained phonetician; see Supplemental Experimental Procedures (section B.4.2) and Figure S1. (C) Statistical parametric map showing the post-DP pattern based on segment prediction error (see Supplemental Experimental Procedures, Section B.4.4), rendered onto an inflated cortical surface of a standard brain in Montreal Neurological Institute space (thresholded at voxelwise p_uncorrected_ < 0.001 for illustration purpose). (D) Estimated source energy averaged over pre- and post-DP time-windows for each condition. Bars represent ± within-participant SE.

By contrast, when epochs were time-locked at UP (i.e., when segment predictions prior to DP are most clearly modified; see Supplemental Experimental Procedures, Section B.1), we observed a reliable post-UP decrease in GFP for trained day 1 versus trained day 2 conditions (Z = −2.07, p < 0.05) (Figure 3A). Thus the pre-DP data are consistent with the segment prediction, but not the lexical entropy account. Section B.2 of Supplemental Experimental Procedures and Figure S1 report a third, alternative “lexical ignition” account that, although also explaining the decreased post-UP MEG signal for day 1 versus day 2 conditions, cannot explain the interaction pattern seen in the post-DP data described below, particularly the greater responses for nonlexical items, i.e., baseline item day 1 and novel/baseline items day 2.

### Effects of Training Day on Post-DP Neural Responses

The lexicality-by-day interaction that was predicted by segment prediction but not lexical entropy accounts was significant in the GFP averaged across the post-DP time-window (Z = 2.55, p < 0.05) (Figure 3A). It was also significant in the sensor-time analyses (Figure 3B) from 280 ms to 350 ms (p corrected < 0.01), with a left temporal focus that resembled the lexicality effect as computed separately for untrained items (see Figure S2) and as observed in previous studies [3, 23]. The simple effect of lexicality (novel versus source items) on GFP was significant for the day 2 condition (Z = −3.61, p < 0.001), but not the day 1 condition (Z = −1.13, p = 0.13). Furthermore, there were significant simple effects of day of training on GFP for novel items and for source items, with a relative increase for day 2 versus day 1 novel items (Z = 1.96, p < 0.05), but a relative decrease for day 2 versus day 1 source words (Z = −2.01, p < 0.05). These simple effects are all consistent with the segment prediction account. The topography and significance (in sensor-time analyses) of these effects are reported in Supplemental Results (Section A.4.2 and Figure S2).

The novelty-by-day interaction was significant in both the GFP (Z = −3.15, p < 0.005) (Figure 3A) and the sensor-time analyses from 100 ms to 500 ms (p corrected < 0.001), again with a left temporal focus (Figure 3B; negative in this case because of the opposite direction of the contrast). The simple effect of novelty (novel versus baseline items) on GFP was significant for the day 1 condition (Z = −3.25, p < 0.005), but not day 2 condition (Z = −0.71, p = 0.48) (see Supplemental Results, Section A.4.2; Figure S2).

In summary, the complete pattern of significant effects in Figure 3A closely matched the predictions of the segment prediction account in Figure 1F. The only exception was the significantly greater MEG GFP for day 1 versus day 2 baseline items (Z = −3.55, p < 0.001), which was not expected (see Discussion).

### Neural Generators of Post-DP Responses

**Figure 4:**
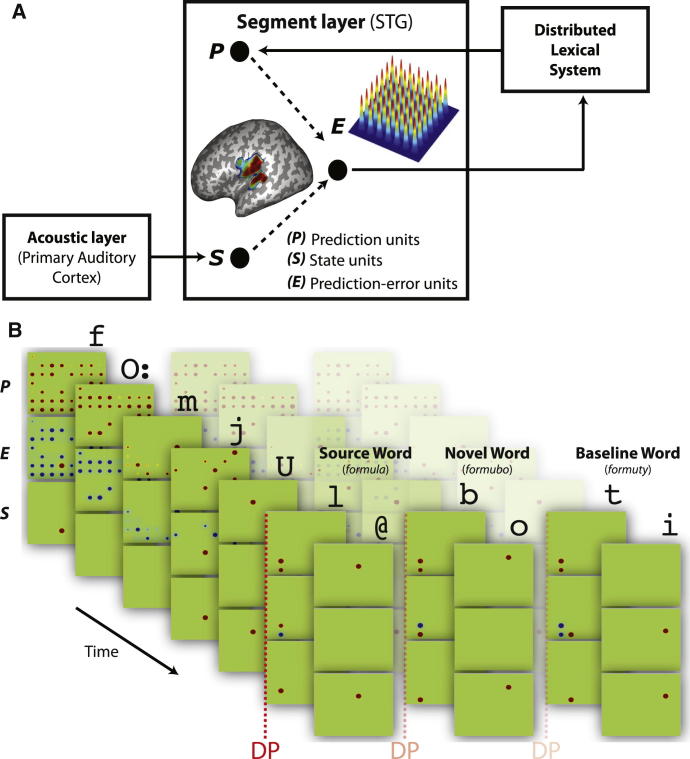
Temporal Predictive Coding Model (A) Speech responsive cortex in the STG has been divided into multiple local patches that code different segments (phonemes here for convenience) illustrated by little Gaussian kernels. Prediction error units in the segment layer encodes the difference between predictions units activated by top-down lexical input and state units modulated by bottom-up activity from sensory acoustic analyses. (B) Illustration of the pattern of activity in the segment layer according to the three types of units (P, prediction units; S, state units, and PE, prediction error units) during recognition of the source word “formula,” novel word “formubo,” and baseline “formuty” after “formubo” has been added to the lexicon. The likelihood density function (the bottom row) represents the level of activity in each state unit coming from the acoustic analysis in lower levels. The prior density (the top row) corresponds to the level of activity in each prediction unit from a lexical system representing the likely identity of the next speech segment predicted from the current speech input using the CELEX database. The difference between the predicted pattern of activity and the pattern arising from sensory evidence determines the level of activity in each prediction error unit (the middle row). Simulation results averaged over all items can be found in Figure S3.

Results of the sensor analyses clearly suggest that changes to the neural response to spoken words and pseudowords reflect computations of segment prediction error rather than lexical entropy. Prediction error is assumed to encode the difference between activity in segment prediction units (derived from a distributed lexical-semantic system) and activity in state units (i.e., sensory evidence) derived from acoustic analysis in lower levels (e.g., primary auditory cortex; see Figure 4). Neural responses linked to this prediction error signal should therefore be localized to neural populations in the STG that have previously been argued to represent the segmental content of speech [18–20]. We therefore estimated the cortical sources of the MEG data during the 100–500 ms post-DP period, and searched for regions that matched the response profile across the six trained (day 1 and day 2) conditions that was predicted by our computational simulation (see Figure 1F; Figure S3). We found two clusters of 1,075 and 717 voxels whose spatial extent survived correction for multiple comparisons. These were spread across the left and right STG, supramarginal gyri, and rolandic operculum (Figure 3C). The largest differences in the response profile for prediction error in Figure 1F arises from lexicalized versus nonlexicalized items. We therefore defined a restricted search volume based on an orthogonal contrast of novel and baseline nonwords versus source words in the untrained condition (this lexicality effect showed good spatial correspondence to prior findings in a meta-analysis of relevant PET and functional magnetic resonance imaging studies [24]; see Figure S2). The peak statistic in both the left (x = −54, y = −12, z = +10, T_(160)_ = 4.7) and right (x = 60, y = −20, z = +12, T_(160)_ = 4.3) STG survived correction for multiple comparisons within this restricted volume (the source energies in left STG peak for each condition, pre- and post-DP, are shown for illustrative purposes in Figure 3D). Thus, source reconstruction further supports the view that MEG signals reflect prediction error at the level of segments, rather than competition at a higher lexical level.

## Discussion

Our results show that differential MEG responses to familiar and consolidated novel spoken words match computations of segment prediction error. These data provide an important additional constraint for neurobiological theories of spoken word recognition. Although most computational theories of spoken word recognition propose competitive evaluation of multiple lexical hypotheses [7–9], we saw no evidence for neural computations correlated with lexical uncertainty. Instead, our results strongly favor computational accounts [10–12] in which the difference between lexical predictions and the current speech input is coded in the STG. Our findings are also consistent with other proposals for predictive coding [25–28], which have become increasingly prominent in various domains of perception including object vision [29] and multimodal integration [30–32]. Our results also complement previous findings on semantic and phonological contextual expectations [33], which appear to have a similar effect on MEG responses as the lexical expectations studied here.

Existing neural simulations of segment prediction for speech have been implemented in connectionist networks [11, 12] that abstract from details of the underlying neurophysiology or in models that have not included lexical-level computations (e.g., [34]). Here, we proposed a neurobiological, temporal, predictive coding model (Figure 4; see Supplemental Experimental Procedures, Section B.3, for further details), in which there are cells in the STG that code the difference between (1) segmental predictions (computed over conventional phonemic transcriptions for convenience) from a lexical-semantic system higher in the linguistic hierarchy and (2) sensory evidence derived from acoustic analysis in lower levels. These cells then project this prediction error to higher levels in the hierarchy to update previously compatible, and hence partially activated, lexical representations. Prediction error cells that feed forward action potentials to higher levels within neocortical hierarchies are the large supragranular, pyramidal neurons [25, 26], which are also believed to be the main contributors to the MEG signal (given that the dendrites of these neurons tend to be aligned). Although we have not fit the precise MEG data in Figure 3 (which would require further assumptions about the dominant current orientation, and precise scaling from simulated activity to magnetic flux/gradient), this neural instantiation of the segment prediction account provides an impressive qualitative fit to the data (Figure S3).

Our model, therefore, mimics probabilistic accounts of spoken word recognition [cf. 8] and the effect of potential lexical competitors [5, 6], without requiring direct lexical-level competition between coactivated units [35]. Instead, according to this predictive coding account, coactivated lexical candidates compete by making incompatible predictions for the speech segments that will be heard next. It is important to note that these “top-down” lexical predictions for upcoming segments do not imply an influence of lexical activity on segment perception through activation feedback [7, 36, 37]. Instead, segment prediction acts to support lexical processing, because segmental predictions that are disconfirmed can be used to directly rule out incompatible lexical hypotheses and accurate segment predictions conversely used to increase the activation of compatible lexical items. Nonetheless, a limitation of our current model is that it does not include a fully specified lexical system; rather, segment predictions were estimated from lexical probabilities derived from the CELEX database. Future work should extend the model to include multiple levels in the speech processing hierarchy.

A related question concerns the nature of the lexical representations. We have confined our experiment and simulations to monomorphemic words. However, response time data suggest that a morphemic entropy measure has the opposite relationship with behavior than does lexical competition (i.e., faster responses for higher entropy; cf. [38]) and further that morphemically structured words evoke differential neural responses time-locked to the onset of inflectional and derivational affixes [39]. This might reflect differences in the predictability of segments in successive morphemes compared to the within-morpheme segment predictions studied in the present article (see [40] for discussion of within- and between-morpheme letter prediction for written language). Further extension of our model may also be necessary to explain the unexpected greater MEG signal for baseline items in the day 1 than day 2 condition. This could reflect, for example, additional interaction with an episodic memory system, which affects processing of baseline items that have not been recently perceived (cf. [24]).

Finally, our inability to detect neural effects predicted by a lexical entropy account does not entirely rule out traditional accounts based on lexical competition (that might occur, for example, in brain regions to which MEG is simply not sensitive). Our point is that we did find evidence consistent with a predictive coding account and that such an account appears able to achieve the same functions without requiring lexical competition. Nonetheless, further evidence and simulations will be required to establish whether or not previously proposed neural correlates of lexical competition (e.g., in frontoparietal regions; cf. [5, 6, 41]), and spoken word recognition [24, 42] can also be explained in terms of predictive coding.

### Computational Simulation Procedures

The computational measures explored here were computed by combining the phonological transcriptions and word frequency measures in the CELEX database [43], for the 216 triplets (source, novel, baseline words) used in this study. We compared predictions when the trained novel word was (day 1) or was not (day 2) included in the lexicon. Because the words used in the experiment are primarily monomorphemic and unlikely to be decomposed during recognition [44], we use the set of uninflected words and confine our analysis to the set of monomorphemic words listed in the database. We further combined frequency measures over homophonic forms of these spoken words. We assume a set of error-free phone recognizers and an optimal recognition process (i.e., an ideal observer). The addition of noise and variability in the speech signal and the suboptimal nature of human speech perception may introduce additional variance, or delays between the speech signal and neural responses, but will not negate the qualitatively different predictions of lexical competition and segment prediction computations. In both cases, we assume that MEG measures the aggregate activity of neural circuits that contribute to segment perception and lexical identification for multiple items. In Supplemental Experimental Procedures (Section B.2), we explore a third potential measure related to activation or ignition of a single lexical item.

#### Lexical Competition

Lexical activation during each segment of a spoken word is expressed as the conditional probability of hearing a word “i” given a speech signal that matches a set “{j}” containing “n” words:

(Equation 1) $$p\left(word_{i}\;|\;speech\right)=\frac{p\left(speech_{i}\;|\;word_{i}\right)\times p\left(word_{i}\right)}{\sum_{j=1}^{n}p\left(speech_{j}\;|\;word_{j}\right)\times p\left(word_{j}\right)}.$$

This conditional probability is computed for each segment in a speech sequence using the phonological transcriptions and word frequencies in CELEX divided by the summed frequency of the set of matching words. Novel words trained on day 1 were assigned a frequency of occurrence equal to the frequency of the source words in the experimental item set; this is the amount of learning assumed for novel words). This equation is identical to that explicitly specified in Bayesian models of word recognition such as Shortlist-B (see Equation 5 in [8]), although these conditional probabilities are also approximated by output activity in localist and distributed neural network models [7, 9]. To take a concrete example, the sequence of segments /k@ptI/ matches just two words, “captain” and “captive” with frequency of 71 per million and 8 per million. Thus, p(captain|k@ptI) = 0.899 and p(captive|k@ptI) = 0.101. We then combine the conditional probability of all activated words using the Entropy measure proposed in Information Theory [45]:

(Equation 2) $$\text{Lexical}\;\text{Entropy}=\sum_{i=1}^{n}-p\left(word_{\text{i}}\;|\;speech\right)\times \text{log}\left(p\left(word_{\text{i}}\;|\;speech\right)\right).$$

#### Segment Prediction Error

Given the set of words in CELEX that match the current speech sequence, and the frequency of occurrence of these words, we can compute the conditional probability of each of the possible segments that could follow the current segment. This is computed using the equation below for the set of n words {j} that are compatible with the current speech sequence and that share the same next segment /i/.

(Equation 3) $$p\left(segment_{i}|speech\right)=\frac{\sum_{j=1}^{n}p\left(segment_{i}|word_{j}\right)\times p\left(word_{j}|speech\right)}{p\left(speech\right)}$$

Therefore, hearing /k@ptI/ (see example above) leads to a prediction that the next segment is either /n/, P(*n*|k@ptI) = 0.899 or /v/ P(*v*|k@ptI) = 0.101 given their relative frequencies above. To turn these predictions into an error measure, we can then compute the summed absolute error between the conditional probabilities for predicted segments and the observed probability of the speech segment. Given an ideal observer, hence error-free segment recognizers, these observed probabilities are one or zero for segments that are heard or absent, respectively.

(Equation 4) $$\text{Segment}\;\text{Prediction}\;\text{Error}=\sum_{i=1}^{n}\left|\left\{1,0\right\}-p\left(segment_{i}\;|\;speech\right)\right|$$

In the example of /k@ptI/, the conditional probabilities of the two next segments (respectively /n/ or /v/) are (0.899 0.101) given the relative frequency of these words. If the final segment of the word “captain” is heard, then the observed probabilities are (1 0), and hence segment prediction error is 0.202.

## Supplemental Information

Document S1. Figures S1–S3, Supplemental Results, and Supplemental Experimental Procedures
