# Supplementary material for: Temporal Predictive Codes for Spoken Words in Auditory Cortex
Source: Curr Biol. 2012 Apr 10;22(7):615–21. doi: 10.1016/j.cub.2012.02.015 (PMC3405519; doi:10.1016/j.cub.2012.02.015)
Supplement: Document S1. Figures S1–S3, Supplemental Results, and Supplemental Experimental Procedures [file mmc1.pdf]

Current Biology, Volume *22*

Supplemental Information

Temporal Predictive Codes

for Spoken Words in Auditory Cortex

Pierre Gagnepain, Richard N. Henson, and Matthew H. Davis

Supplemental Inventory

1. Supplemental Figures and Tables

Figure S1, related to Figure 1

Figure S2, related to Figure 3

Figure S3, related to Figure 4

2. Supplemental Results

3. Supplemental Experimental Procedures

4. Supplemental References

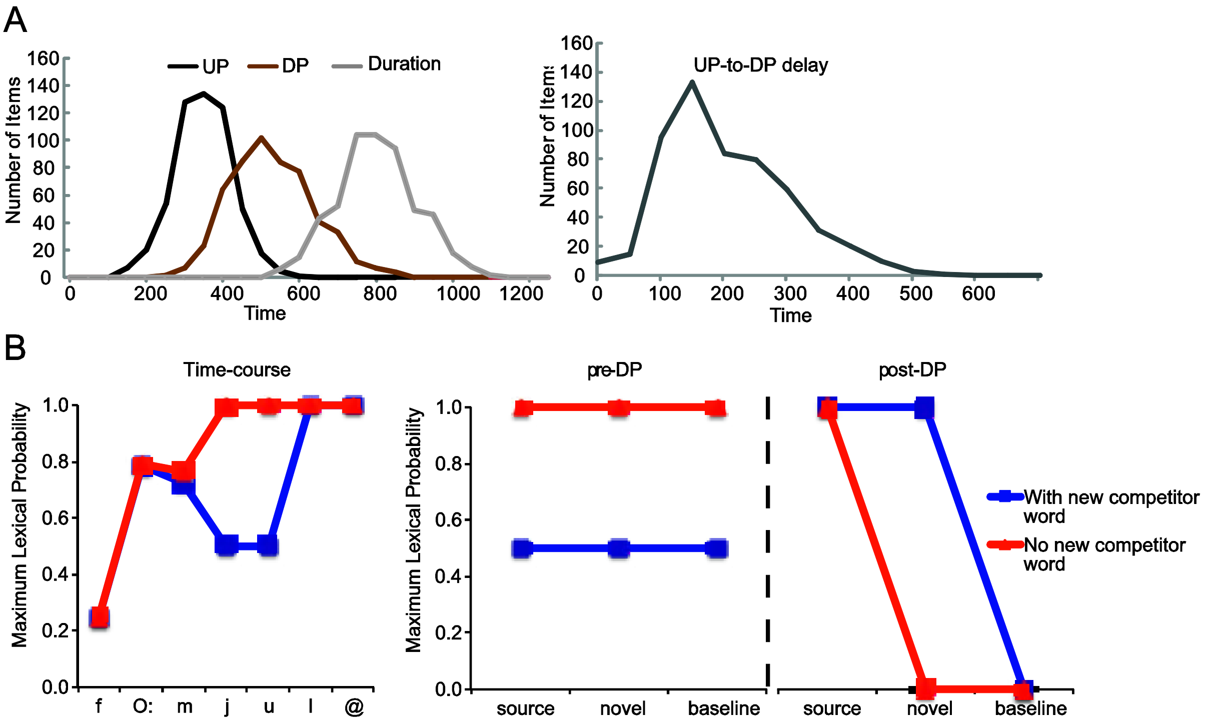

Figure S1. Related to Figure 1

(A) Uniqueness point (UP), Deviation point (DP), and duration distributions of the spoken words used in this experiment. The distribution of these time lags shows that more than 85 % of our items have a UP-to-DP delay of less than 400 ms, indicating that competition from a consolidated spoken word should be most apparent within a -400 ms to 0 ms time window (0 ms being the DP; see Figure 2A). However, given known delays in cortical transmission of sensory information of approximately 100ms (i.e. when one typically observes the first significant peak evoked response in auditory cortex, Davis and Zerlin, 1966), we shifted this pre-DP time-widow of interest to -300 to 100 ms. Furthermore, we only included in the UP-locked analyses items with UP-to-DP delays greater than 100 ms in order to not include any post-DP effect (note, however, that the results were essentially the same when all items were included).

(B) Computational simulations of lexical ignition. The impact of an additional lexical candidate (*formubo*) on the recognition time-course of the familiar word *formula* /fO:mjul@/, plotted as for Figure 1C/D. Experimental predictions for neural correlates for pre-DP and post-DP responses in the 6 critical conditions in our experiment, plotted as in Figure 1E/F.

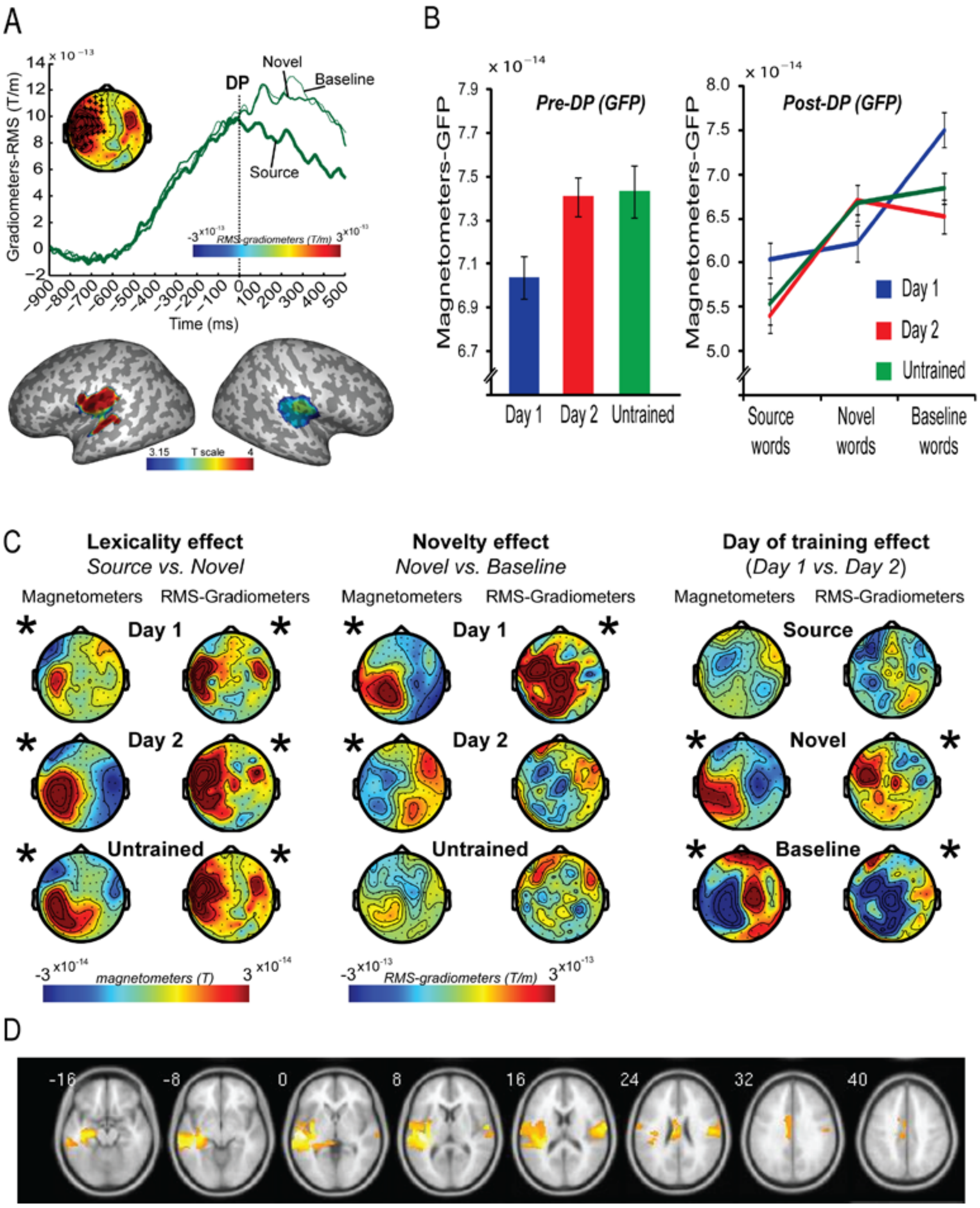

Figure S2. Related to Figure 3

(A) Time-course plots of significant RMS-gradiometers sensors (marked by a cross in the scalp topography) for the *Lexicality effect* in the Untrained condition. Statistical parametric map showing the *Lexicality effect* in the Untrained condition rendered onto an inflated cortical surface of a standard brain in MNI space (thresholded at voxelwise P-uncorrected < .001 for purposes of illustration).

(B) Magnetometers Global Field Power (GFP; i.e. RMS across all sensors) averaged over the pre-DP time-window (UP-locked epochs) and the post-DP time-window (DP-locked epochs) for the Trained Day 1 (blue line), Trained Day 2 (red line) and Untrained (green line) conditions. Error bars show +/- within-participant standard error.

(C) Magnetometer and RMS-gradiometer topographies for simple effects composing the interactions of interest shown in Figure 3. Significant effects in Sensor -Time analyses are marked by an asterisk.

(D) Statistical parametric map of the source reconstruction based on the combined gradiometer and magnetometer data (Henson et al., 2009) showing the expected pattern of prediction error post-DP (see Supplemental Experimental Procedure, Section B.4.4) rendered onto a standard brain in MNI space (thresholded at voxelwise P-uncorrected < .001 for purposes of illustration).

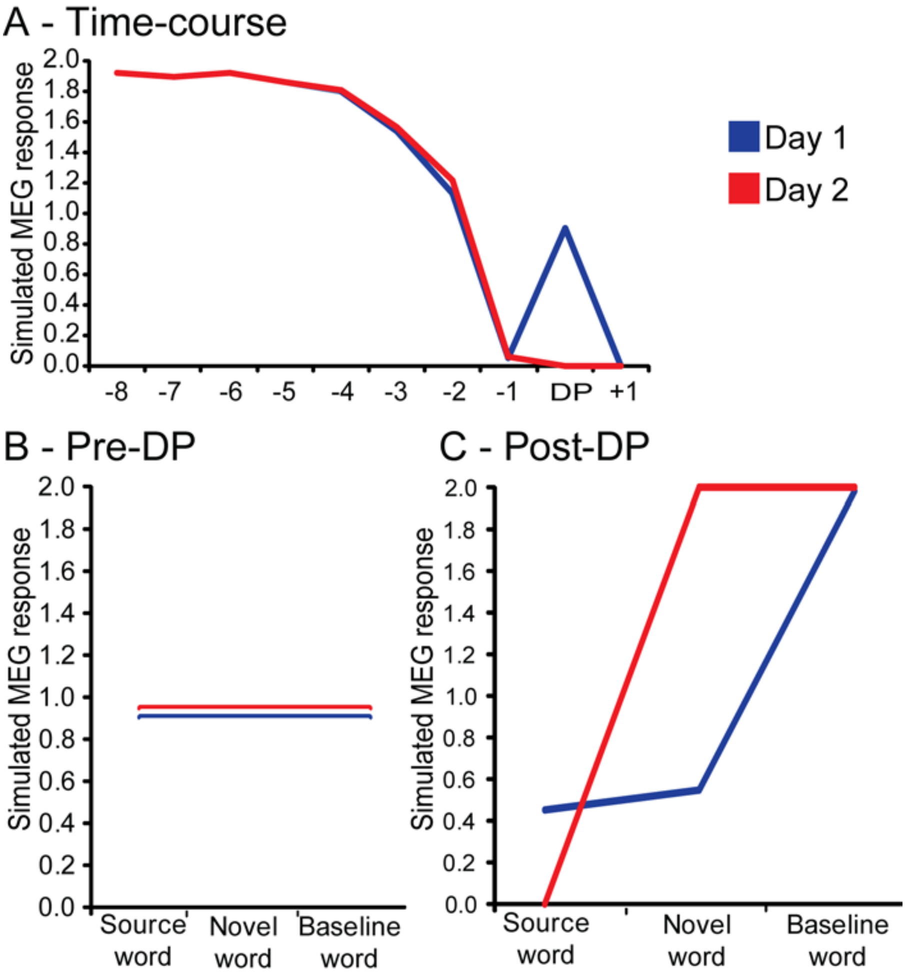

Figure S3. Related to Figure 4

(A) Simulated MEG response as computed by the neural instantiation of the temporal predictive coding account. The three plots represent the prediction error (i.e. the mean sum of absolute activity in the segment layer) averaged over all the items in the item set, for (A) the time-course of recognition of a Source word (e.g. *formula*) relative to the deviation point segment (DP) as a function of whether a new competitor is learned on Day 1 (blue line) or on Day 2 (red line).

(B) Prediction error averaged over the pre-DP segments for Source, Novel and Baseline item.

(C) Post-DP prediction error plotted as in (B). See also Figure 1E/F for comparison.

Supplemental Results

Magnetometer Global Field Power (GFP)

Pre-DP Analyses

The GFP for the magnetometers showed no reliable differences [Z = -1.34, P = .18] between trained Day 1 vs. trained Day 2 conditions when averaged over the pre-DP time-window of interest (-300 ms to 100 ms; see Supplemental Experimental Procedure, Section B.4.4, and Figure S1). In fact, if anything, there was a numerical trend in the opposite direction to that predicted by lexical entropy accounts, i.e., greater GFP for trained Day 2 vs. trained Day 1 conditions.

Careful inspection of the segment prediction simulations show that phonemic predictions prior to DP are most clearly modified at the original UP (i.e. the /j/ of *formula*) and may explain this tendency for decreased pre-DP responses with consolidation. To confirm this prediction, we ran a further analysis of the pre-DP data, using epochs time-locked to the UP (i.e. post-UP analysis). If as shown in the simulations that the small decrease in pre-DP prediction-error is locked to the UP then we should now observe this effect 100 ms after the actual UP (taking into account the auditory delay; see Supplemental Experimental Procedure, Section B.4.4). Analysis of the magnetometers GFP indeed showed a reliable decrease for trained Day 1 vs. trained Day 2 conditions when averaged over 100 ms to 200 ms (to exclude any post-DP effect) after the UP ([Z = -2.35, P < .05]; see Figure S2). Note that once again to exclude any post-DP effect, this analysis was performed only on items with a UP-to-DP delay greater than 100 ms (see Figure S1).

Post-DP Analyses

In this section, we assess the predicted neural profile of prediction error using two interactions described in Figure 1F: (1) the *Lexicality-by-Day interaction* and (2) the *Novelty-by-Day interaction*. The *Lexicality-by-Day interaction* [(Source word Day 1 - Novel word Day 1) – (Source word Day 2 - Novel word Day 2)] was significant in the magnetometers GFP averaged over the post-DP time-window of interest (see Supplemental Experimental Procedure, Section B.4.4, and Figure S2) [Z = 2.52, P < .05]. To confirm that this interaction was driven by lack of consolidation of Novel trained Day 2 items, simple effects of *Lexicality* were tested separately on the GFP for Day 1 and Day 2 conditions. The *Lexicality effect* (Novel vs Source items) in the Day 2 conditions was significant [Z = -3.32, P < .001], but failed to reach significance in the Day 1 condition [Z = -1.1, P = .22].

The *Novelty-by-Day interaction* [(Novel word Day 1- Baseline word Day 1) – (Novel word Day 2-Baseline word Day 2)] was significant in the magnetometers GFP average averaged over the post-DP time-window of interest ([Z = -2.94, P < .005]; Figure S2). To confirm that this interaction was driven by consolidation of the Novel trained Day 1 items, simple effects of *Novelty* were tested separately in the GFP for Day 1 and Day 2 conditions. The *Novelty effect* (Novel vs. Baseline items) was significant in the Day 1 conditions [Z = -3.28, P < .001] but not in the Day 2 condition [Z = -0.76, P = .44].

The presence of these two interactions provides evidence that training and overnight consolidation but not training alone led to the lexicalisation of the novel spoken words in the Day 1 condition. The direction of these interactions was exactly as expected by an account of spoken word recognition in which neural responses to incoming segments reflect prediction error. We observed a marginal trend for the expected increase in magnetometers GFP for Day 1 Source words relative to Day 2 Source words [Z = -1.67, P = .09]. Moreover, we also observed the expected decrease for Novel word Day 1 relative to Novel word Day 2 [Z = 2.3, P < .05]. However, we again saw the same unexpected difference for Baseline items characterised by greater GFP for Day 1 than Day 2 conditions [Z = -2.64, P < .01] that we observed for gradiometers (see Figure 3A and Figure S2B).

Additional Sensor-Time Analyses

Pre-DP analyses

Results of the Sensor-Time analyses of the DP-locked epochs showed a reliable difference for the trained Day 1 condition in magnetometer ERF (P-corrected < .05) suggesting that MEG effects were localised to a few sensors and hence attenuated when data was combined over sensors in the GFP. However, this pre-DP difference between Day 1 and Day 2 items failed to show a reliable difference for RMS-Gradiometers as mentioned in the main text (P-corrected = .13). Similar findings were found pre-DP for Sensors-Time analyses performed on UP-locked epochs. A marginal difference was observed between Day 1 and Day 2 for ERF magnetometers (P-corrected = .09) while this effect failed to reach significance for ERF RMS-gradiometers (P-corrected = .15).

Post-DP Analyses

The *Lexicality-by-Day interaction* failed to survive correction in the Sensor-Time analysis of the magnetometers data (P-corrected = .15) though it was significant for RMS-gradiometers from 280 ms to 350 ms as reported in the main text (P-corrected < .05). A significant *Lexicality effect* in the Day 2 condition was observed for both magnetometers from 100 ms to 500 ms (P-corrected < .001) and RMS-gradiometers from 120 ms to 370 ms (P-corrected < .001). Despite the presence of a significant *Lexicality-by-Day interaction*, we also observed in these Sensor-Time analyses a residual *Lexicality effect* in the Day 1 condition from 100 ms to 300 ms for magnetometers and RMS-gradiometers (P-corrected < .05). This residual *Lexicality effect* for items trained on Day 1 condition reflects a difference between words learned on the previous day and words in participants' long term vocabulary; this might be attributed to the lack of conceptual or semantic information and/or that these items are not of equivalent frequency or familiarity as the corresponding source words.

We also observed a significant *Novelty-by-Day interaction* from 100 ms to 500 ms for both magnetometers (P-corrected < .001) and RMS-gradiometers (P-corrected < .001). The expected *Novelty effect* was present in the Day 1 condition from 100 ms to 500 ms for both magnetometers (P-corrected < .001) and RMS-gradiometers (P-corrected < .001). However, a significant *Novelty effect* was also observed for Day 2 items (not significant in the GFP analyses) from 220 ms to 500 ms for magnetometers ERF (P-corrected < .001). Such effect was not reliable for RMS-gradiometers (P-corrected = .22). As it can be seen from the gradiometers GFP reported in Figure 3B, this effect goes in the reverse direction of the *Novelty effect* in the Day 1 condition. This might reflect an influence of recent learning on post-DP responses that was not expected such as inhibition or interference between trained Novel words and nonword Baseline items.

Finally, we also compared trained Day 1 and trained Day 2 conditions for each condition separately (i.e. Source, Novel and Baseline words). Regarding Source words, Sensor-Time analyses showed no significant difference for both magnetometers (P-corrected = .6) and RMS-gradiometers (P-corrected = .15). The Day of training effect was significant for Novel words for magnetometers from 250 ms to 500 ms (P-corrected < .05) and for RMS-gradiometers from 260 ms to 330 ms (P-corrected < .05). Lastly, Baseline items were also associated with an unexpected significant difference for both magnetometers (P-corrected < .001) and RMS-gradiometers (P-corrected < .001) from 100 ms to 500 ms. The topography and significance (in Sensor-time analyses) of these effects are shown in Figure S2.

Supplemental Experimental Procedure

Distinguishing Lexical Competition and Segment Prediction

We use an artificial word learning method to distinguish two different computational mechanisms that have been proposed to be key to the rapid and efficient recognition of spoken words. Specifically, we contrast computations of lexical competition (quantified using a lexical entropy measure), and segment prediction (quantified as the discrepancy between heard and lexically expected segments in speech). The two computational measures are reported in detail in the Computational Simulations section of the main manuscript. Neural correlates of these two computations are difficult to distinguish for natural language stimuli since segmental and lexical uncertainty change in parallel during perception of spoken words. One illustration of this is that for the set of 216 source words used in the experiment, the segment-by-segment correlation between lexical entropy and segment prediction error is extremely high ($r= 0.602$, $P<.001$). It would be difficult to distinguish such highly related computational signals even if these segment-by-segment measures could be easily aligned to the speech signal for each spoken word. These two measures remain correlated when they are averaged over the entirety of a spoken word ($r= 0.473$, $P<.001$) and so it might also be difficult to distinguish between these measures using measures like fMRI which record an aggregate neural responses for an entire spoken word.

However, by manipulating the set of spoken words that participants know at the time of testing (through training on artificial vocabulary items) we can create stretches of speech during a spoken word when these two computational measures make opposite predictions – i.e. situations in which lexical uncertainty is high but segment prediction error is low and vice-versa.

Lexical entropy quantifies the degree of lexical uncertainty at specific points in a speech sequence. All other things being equal, lexical entropy will be maximal at word onset (when the entire lexicon is partially activated) and then declines over time as fewer words match the speech signal. When only a single lexical item remains, its conditional probability will be one and lexical entropy will be zero. Similarly, when a pseudoword is presented, an ideal observer model will rule out all known words and lexical entropy will again be zero. In order to compute the conditional probability and lexical entropy for novel words that have been integrated into the lexicon after consolidation (trained Day 1 condition), we need to supply a phonological transcription for our recordings, and assign them a frequency of occurrence. This frequency value is a direct measure of how well-learned the novel words are by comparison with real words which have a frequency of occurrence based on corpus counts, and may influence the quantitative scaling of the computed effect. For this purpose, we assume that novel words learned on Day 1 have a frequency equivalent to the frequency of the 216 source words in the experimental item set.

Segment prediction error quantifies the mismatch between segment conditional probability (again computed using word frequency and phonological transcriptions) and the observed probability of the speech segment (which can be 0 or 1 according to an ideal observer). In contrast to the conditional probability of a word given a speech input in the case of lexical competition (equation (1) in the Computational Simulations section), segments that are predicted on the basis of multiple words become more likely (equation (3) in the Computational Simulations section). Hence increased lexical uncertainty may not lead to increased segment prediction error if several lexical items all predict the same upcoming segment. However, lexical items are ordinarily distinguished by containing different subsequent phonemes. Hence, for speech sequences taken from natural language, segment prediction error will almost inevitably become lower (i.e. predictions are more accurate) as lexical uncertainty declines.

One key difference between segment prediction error and lexical entropy is that whereas lexical entropy goes to zero after the DP of a nonword (since no lexical item matches the input), segment prediction error will increase at or after the DP of a pseudoword since the phonemes heard were not predicted. Furthermore, segment prediction error at the DP will decrease once a

pseudoword has been learned and consolidated, and to a degree that depends on the frequency of the learned pseudoword and the number of candidate words that match the previous speech.

For example, before *formubo* is consolidated, the conditional probabilities of /l/ and /b/ when hearing the initial portion of *formula* (i.e. p(l|fO:mjU) and p(b|fO:mjU) are respectively [1 0], but after consolidation of *formubo* change to [.5 .5] (assuming equal frequency for *formubo* and *formula*) thereby reducing prediction error on hearing /b/ from 2 to 1 (given equation (4) in Computational Simulations section). If an unrelated word is heard (e.g. the baseline item *formuty*, containing the consonant /t/) then prediction error will be 2 prior to consolidation of *formubo*, and 2 after consolidation.

Note that, to map from the local prediction error for each segment to the gross MEG GFP, we used the sum of absolute values of prediction error over segments. Given that the sign of the error can be positive or negative this cannot easily be related to the MEG signal (it will depend on the direction of current and orientation of dendritic currents whether positive and negative signals cancel out or summate). We also computed the sum of squared prediction errors instead: the results were identical, except that smaller signal was predicted for consolidated (Day 1) Baseline items (1.5) than the other Baseline conditions, the opposite of what was found. Given that the sum of absolute values also failed to predict this increased response for Baseline items (see main text), we used this measure for simplicity.

Opposite changes in segment prediction error and lexical entropy are also seen prior to DP. Prediction error will decrease with consolidation if the novel word predicts the same segment as contained in other lexical items. For instance, the likelihood of hearing /j/ given the initial segments /fO:m/ is increased following consolidation since both *formula* and *formubo* predict the same subsequent segment (i.e. /j/). Thus, whereas consolidation of *formubo* increases lexical uncertainty between the UP and the DP of *formula*, prediction error is decreased since both lexical items predict the same segment. In the example given above however, prediction error is zero for segments between the UP and DP (since p(U|fO:mj) = 1, i.e. there is a unique potential upcoming phoneme) and hence we only see a reduction in prediction error at the UP itself in the context of multiple phonemic predictions (the segment (/j/), corresponding to the original UP of *formula*, has an increased conditional probability with respect to other phonemes). For this reason, we expect subtle decreases in the neural response in the Day 1 condition before the DP and time-locked to the UP.

These opposite changes to lexical entropy and segment prediction error pre-DP and post-DP are maximised by our experimental materials. We deliberately chose words with an early UP, and added novel competitors that make the DP as late as possible. In this way, we gain the maximum temporal separation of neural responses that are pre- and post-DP (see Figure S1 for the timing of UP and DP segments in our set of items). However, we note that a more limited form of this dissociation is possible in real words – despite the correlations between lexical entropy and segment prediction error reported earlier. For instance, during the spoken word *"succulent"* (/sVkjUl@nt/, segment prediction error goes to zero following /sVkj/ since all the other matching competitors (words like *"succubus"*) predict the same segment /U/. However, there are a limited number of these items in natural language, and only a brief period during these words in which segment prediction error and lexical entropy dissociate. The unique strength of our experimental design is that it creates a large number of items in which there is the maximum possible dissociation between measures of lexical entropy and segment prediction error.

Lexical Ignition—an Alternative Account of the Link between Neural Activity and Spoken Word Recognition

We have described two possible computational accounts in which neural activity is predicted to decrease as more of a spoken word is processed, either due to a reduction in lexical competition (quantified using entropy), or due to increased accuracy of sub-lexical predictions (quantified using segment prediction error). However, another possibility is that neural responses measured using

MEG are associated with the activation of word-specific representations. One possible mechanism for this is found in neural models of spoken word recognition proposed by Garagnani and colleagues (Garagnani et al., 2008; Garagnani and Pulvermuller, 2011), which have been linked to MEG responses for words and pseudowords in mismatch negativity studies (Garagnani et al., 2009). In this account, words are represented in the form of Hebbian cell assemblies that are activated ('ignite') when appropriate input is provided. This therefore leads to the prediction that neural activity will be maximal when the speech signal matches one word – i.e. lexical ignition occurs when the conditional probability of one word is high. At present, however, there are no simulations of this account that make explicit predictions concerning the response to sequences of speech sounds that unfold over time and hence no way in which we can use this model to derive neural predictions directly from simulations.

There are, however, ways in which we can derive predictions from this account using an assumed relationship between neural activity and the conditional probability of words given the current speech input (similar to that used for lexical competition accounts). For instance, we could assume that a threshold is applied to lexical activity such that lexical ignition and word recognition occurs when a single candidate becomes sufficiently probable. However, it might be unclear what probability threshold is required to avoid false identification. A second alternative is to combine probabilities over multiple lexical candidates so that lexical ignition is proportional to the difference in probability between the most probable word and other candidates (i.e., use a relative rather than absolute threshold). This can be expressed in a form that is numerically opposite to lexical entropy, so as to create a measure that is maximal when one word is highly probable. The simplest procedure is to assume that neural activity reflects the probability of the most likely word at any point in time. This is the assumption that we make in the simulations reported in Figure S1, (graphs formatted as in Figure 1B-F). Here we report the conditional probability (as in equation 1 of the paper), for the most probable word at every point in the sequence. The other two possible methods (thresholding, or combining probabilities) lead to essentially the same predictions.

Note that while the profile depicted simulates the MEG results observed for the pre-DP period, the post-DP response profile is opposite to the MEG results. Lexical ignition predicts maximum activity for source words and consolidated novel words, and little or no activity for unconsolidated novel words and baseline items.

Temporal Predictive Coding (a Neural Illustration of Segment Prediction Error)

This section provides an illustration of how the pattern of electrophysiological responses in the superior temporal gyrus (STG) might be simulated within a predictive coding framework. We make the following assumptions:

1. The STG contains a layer of sparse representations (e.g. in cortical columns) for each phoneme in the language. These representations may be topographically-organised (based on acoustic similarity and confusability), but for simplicity, we model the 56 phonemes in our stimulus set by an arbitrary grid of 7x8 localist representations (units). This STG layer is assumed to be connected to a "lower-level" acoustic layer (e.g. in primary auditory cortex), and a "higher-level" lexical layer (e.g. in lateral temporal regions surrounding the STG).

2. Each unit contains three types of cell (likely in different cortical layers; Friston, 2005): (i) cells that code the "state" or input from the acoustic layer, (ii) cells that code the prediction from the higher, lexical layer, (iii) cells that code the "prediction error" (i.e. the difference between state and prediction cells). Let the activity of each of these cells in the *j*th STG unit during each speech segment *i* be $S_j(i)$, $P_j(i)$ and $E_j(i)$.

3. Assuming perfect acoustic processing, and that segment *i* corresponds to phoneme *q*, then let $S_j(i)=1$ for $j=q$, and $S_j(i)=0$ otherwise.

4. The activity in the prediction units for segment *i* is based on the prior probability of each phoneme, given the previous segments, based on input from the lexical system, as in Equation 3 in Experimental Procedure, where this conditional probability is computed from the CELEX lexical database, as explained in Section 2 above.

5. The activity of the *j*th prediction error cell for segment *i* is simply $E_j(i) = S_j(i) - P_j(i)$. Assuming that the MEG signal is related to these error cells (if they correspond to the large pyramidal cells that provide input to the layer above, Friston, 2005), and integrated over all such cells in each layer (again assuming the sign is irrelevant), then the MEG signal for segment *i* is $\sum_{j=1}^{56} | E_j(i) |$.

To confirm that these computations are similar to the segment prediction error simulation reported in the main text, we ran this simulation for all of the 216 triplets (Source, Novel, Baseline words) used in this study, in both situations when the trained novel word was (Day 1) or was not (Day 2) included in the lexicon. As for the initial simulation, we assume that novel words learned on Day 1 have a frequency equivalent to that of the source words, though in this case, we assigned the frequency of all consolidated novel words to be 3 / million (this is the harmonic mean of the frequency of the 216 source words in the experimental item set). We then averaged over all the experimental item triples to produce the results in Figure S3. Note that in Figure 4, we used a set of Gaussian kernels to visually represent the pattern of activity. However, the outcome will be exactly the same providing the total activity within each type of cell is normalised to one at each time-point to correspond to probabilities (the absolute value of prediction-error will differ, not the relative pattern).

Supplemental Procedure

Participants

24 right-handed English native speakers (10 males) aged between 20 to 30 years (M = 23.7; S.D. = 2.3) were paid to take part in the study. They had no reported history of neurological, medical, speech or hearing disorders. The project was approved by the Cambridge Psychology Research Ethics Committee and all participants gave written consent. Three participants were excluded from subsequent analyses due to falling asleep during recordings (1 participant) and failure to estimate head position indicators and compensate for head movements (2 participants). Subjects were instructed not to consume psychostimulants, drugs, or alcohol prior to and throughout the experimental period. Quality of sleep (mean duration = 8.2 hr ; SD = 1.3) between the first initial training on Day 1 and the final test on Day 2 was assessed using the St. Mary's Hospital sleep questionnaire (Ellis et al., 1981) and all participants reported having slept well or very well.

Stimuli

180 pairs of novel words (e.g. "*formubo/formuty*") were derived from 180 real words (e.g. "*formula"*). The majority (75%) of the novel words were created by substituting final segments of their source words (e.g. "*formubo*" for "*formula*", cf. Gaskell and Dumay, 2003) and 25% had additional segments added to the source words (e.g. "*bingoak*" for "*bingo*", cf. Dumay and Gaskell, 2007). The 2 versions of each of the novel spoken words had the same structure but included different changes to the final segments. From this set of 540 items (360 novel words, 180 real words), three matched subsets of 60 triples (1 source word and 2 novel words), were created that would be assigned to the different experimental conditions in a counterbalanced fashion (see Figure 2A). The 3 lists were matched on various criteria, including the proportion of pseudowords created by substituting vs. adding segments, the CELEX frequency of the source words (Baayen et al., 1996), number of segments (5 to 8; average = 6.3 segments), number of syllables (bi-/tri-syllabic; average = 2.5 syllables), their uniqueness point (UP) as computed from CELEX (average = 3.98 segments), their

terminal set (i.e. number of words corresponding to the pre-uniqueness point segment; average = 22), their number of post-UP segments (average = 2.3 segments), and the number of segments between UP and the divergence point (DP) between the source and novel words (average = 1.2 segments). In addition to these three lists of 60 triples, three further sets of 12 triples (i.e. 36 source words, and 72 novel words, 108 items in total) were created in the same way and used as targets for the pause-detection task (see Section B.4.3 below).

The 648 spoken words were recorded onto a high-quality digital recorder (Marantz, Japan) by a male native speaker of British English in a soundproof booth at a sampling rate of 44.1 kHz with care taken to ensure that the shared segments of the spoken words and pseudowords were pronounced identically – in terms of their pitch accent, stress pattern, etc. However, naturally occurring forms of anticipatory coarticulation will still be found in these recordings. Sound files were digitally transferred to a computer, divided into single sound files using Adobe Audition software (San Jose, CA), and trimmed to length. The mean duration of these sound files was 778 ms (ranging from 507 ms to 1100 ms), producing an average speech rate of 3.25 syllables/second.

Two trained phoneticians listened to each of the recordings and marked in the speech signal when deviating speech segments could be identified for each source, novel and baseline spoken words (i.e. the Deviation Point, the onset of the final consonant /l/ for *formula*, /b/ for *formubo* and /t/ for *formuty*), and the segment that made each item unique from all other known words (i.e. the Uniqueness Point, in this case the vowel /j/ which mismatches with *format*, *formal*, etc). DP and duration distributions of the novel spoken words, as well as the distribution of UP-to-DP time lag, are reported in Figure S1. In marking the onset of the deviating and lexically unique segments were informed that they should mark the earliest point at which the stimuli diverged phonetically and hence should take into account reliable coarticulatory cues to distinguish between source and novel words where these are present.

In linking estimates of lexical entropy and segment prediction error to the speech signal we used the acoustic onset of critical segments in the speech signal as the time-point at which event-related MEG signals will be aligned. This underestimates the timing of critical events – acoustic information after this onset will be required for identification – however, this is a conservative approach for assessing whether information before or after the uniqueness and deviation points is reflected in neural responses. Since these phoneticians were naïve to the purpose of the experiment, and critical materials were counterbalanced over conditions, errors in the identification of these acoustic landmarks could not contribute to the results observed.

Finally, a190 ms pause was inserted into the 108 items to be used the pause-detection task approximately 1 or 2 phonemes before the DP.

Tasks and Procedures

Learning Task

The experiment was performed in two sessions on two consecutive days. On Day 1, participants undertook 1080 trials of a phoneme-monitoring test with each of 60 novel words (one pseudorandomly selected from each *formubo/formuty* pair) presented 18 times in a training session lasting approximately 40 min. They were also trained for 216 additional trials (18 presentations / item) on 12 novel words that would be used as targets for the pause detection task. On Day 2, approximately 24 hours after the first session, participants performed the same phoneme monitoring task with a new list of 72 novel words (60 test items plus 12 items to be used in pause detection).

The training procedure used was similar to that described in Davis et al. (2009) - Prior to each block of 72 trials (60 critical novel words + 12 subsequent pause-items), a visual display signalled a target phoneme that participants should listen for (/p/, /t/, /k/, /l/, /n/, /s/, /b/, /d/, /dZ/, used twice each). Participants were required to indicate with a button-press when they detected the

target phoneme and had 1.9 sec to respond. Item order was randomised across blocks and each block was repeated 18 times.

Subject Preparation for MEG

Approximately 60 min after the end of the second training session, participants were prepared for MEG. This included 3D digitisation of their fiducials and headshape data, and also attachment of 70 EEG electrodes (because of relatively high levels of noise in the EEG data, they are not reported here). A 3D digitizer (Fastrak Polhemus Inc., Colchester, VA) was used to record the locations of Head-Position Indicator (HPI) coils and approximately 50–100 'head points' along the scalp, relative to three anatomical fiducials (the nasion and left and right pre-auricular points).

Pause-Detection Task (MEG Recording)

The MEG data were acquired while participants performed a pause-detection task (Mattys and Clark, 2002) on: 1) Novel words trained on Day 1 and their neighbouring Source and Baseline words (Day 1 condition), 2) Novel words trained on Day 2 and accompanying Source/Baseline words (Day 2 condition), and 3) a further set of Novel, Source and Baseline words (Untrained condition), resulting in a 3 (Learning: Day 1, Day 2, Untrained) x 3 (Item type: Source, Novel, Baseline words) factorial design (see Figure 2A). The three lists of 60 word triples were counterbalanced across conditions and participants, and the specific item assigned to the novel or baseline word conditions (e.g "*formubo*" or "*formuty*") was also counterbalanced. Items trained for use in the target detection condition were assigned to the 9 conditions and counterbalanced in the same way. These 108 items (12 items in each of 9 conditions) were presented with a pause during the MEG recording sessions and randomly intermixed with the 540 items in the 9 conditions of interest.

MEG recordings were divided into 3 sessions each of about 11 min containing one item from each of the 216 triples (i.e. only one of the source/novel/baseline items), with equal numbers coming from the 9 experimental conditions. Stimuli were presented with a variable inter-stimulus interval between 1850-2150 ms. The order in which items from each triplet were presented was determined pseudo-randomly and matched across learning conditions, such that within-test learning could not explain results obtained. The task of the participants was to press a button with their left hand as quickly and accurately as possible when they detected a pause inserted in a spoken item.

The task of the participants was to press a button with their left hand as quickly and accurately as possible when they detected a pause inserted in a spoken item. However, given the small numbers of these items (12 responses/condition/participant), we also used a delayed repetition task (cf. Davis et al., 2009) to examine effects of consolidation and training on the three sets of 60 novel spoken words (Day 1/Day 2/Untrained).

Repetition Task

This test assessed whether the speed of speech production is enhanced for items trained on the same- or previous-day. All 180 novel words (60 Day 1/Day 2/Untrained) were presented in random order, followed, after a variable stimulus onset asynchrony ranging from 1550-2550ms, by a 380ms tone that cued participants to say the word aloud as quickly as possible (see Figure 1B). Vocal response latencies were recorded from the beginning of the cue tone using the E-Prime voice key. However, due to technical problems, data from 2 participants were lost.

MEG Recording and Analyses

MEG data were collected with a VectorView system (Elekta-Neuromag, Helsinki, Finland), containing a magnetometer and two orthogonal, planar gradiometers located at each of 102 positions within a hemispherical array situated in a light Elekta-Neuromag magnetically-shielded room. The position of

the head relative to the sensor array was monitored continuously by feeding sinusoidal currents (293–321 Hz) into four Head-Position Indicator (HPI) coils attached to the scalp. Vertical and horizontal EOG were also recorded. All data were sampled at 1 kHz with a band-pass filter from 0.03–330 Hz. External noise was removed from the MEG data using the temporal extension of Signal-Space Separation as implemented with the MaxFilter software, Version 2.0 (Elekta-Neuromag). The MEG data were compensated for movement every 200 ms. Manual inspection identified some bad channels (0-3 channels across participants) which were recreated by MaxFilter. The data were down-sampled to 250 Hz (i.e. 4 ms sampling), using an anti-aliasing lowpass filter cutoff of 111 Hz. We only report data from the gradiometers in the main paper, because they contain greater spatial information (for superficial sources), are less sensitive to deep brain sources (which can be desirable for the largely superficial brain regions associated with speech perception) and are more amenable to topographic analysis (though very similar results are found in the magnetometer data, as reported in Supplemental Results, Section A.4).

Event-Related Fields (ERF)

Data were read into SPM8 (http://www.fil.ion.ucl.ac.uk/spm/), low-pass filtered to 20 Hz in forward and reverse directions using a 5th-order Butterworth digital filter, and then epoched from -900 to 500 ms locked to DP. The mean baseline computed from -900 ms to -500 ms (before word onset) was removed from epoched data and robust averaging applied to minimise non-phase-locked artefacts. For sensor-time analysis of planar gradiometer data, the Root-Mean Square (RMS) of the two orthogonal gradients was computed to estimate the gradient magnitude at each sensor location (this step was not performed for the source localisation). The exact same procedure was applied to UP-locked data with epoch of -700 ms to 500 ms and baseline correction time-window -700 ms to -400 ms. The time window of interest for pre-DP comparison was -300 to 100 ms for DP-locked epochs and 100 ms to 200 ms for UP-locked epochs given the 100ms delay in cortical transmission of sensory information (Davis and Zerlin, 1966; see also Figure S1). Furthermore, we only included in the UP-locked analyses items with UP-to-DP delays greater than 100 ms (Figure S1) to exclude any post-DP effect in this analysis (note, however, that the results were exactly similar when all items were included). The time-window for the post-DP comparisons was 100 ms to 500 ms based on the time range showing a significant *Lexicality effect* for Untrained items (see Figure S2).

Source Reconstruction

A structural MRI image for each participant was obtained on a 3T MR system (Siemens, Erlangen, Germany) using a T1-weighted GRAPPA 3D MPRAGE sequence (TR=2250ms; TE=2.99ms; flip-angle=9 degrees; acceleration factor=2) with 1mm isotropic voxels. This image was spatially normalised to grey matter, white matter and CSF segments of an MNI template brain in Talairach space. The inverse of this spatial transformation was then used to warp a cortical mesh from that template brain back to each subject's MRI space (Mattout et al., 2007). The resulting 'canonical' mesh was a continuous triangular tessellation of the grey/white matter interface of the neocortex (excluding cerebellum) created from a canonical T1-weighted MPRAGE image in MNI space using FreeSurfer. The surface was inflated to a sphere and down-sampled using octahedra to achieve a mesh of 8196 vertices (4098 per hemisphere) with a mean inter-vertex spacing of ~5 mm. The normal to the surface at each vertex was calculated from an estimate of the local curvature of the surrounding triangles. The same inverse-normalisation procedure was applied to template inner skull, outer skull and scalp meshes of 2562 vertices.

The MEG sensor positions were projected onto each subject's MRI space by a rigid-body coregistration based on minimising the sum of squared differences between the digitised fiducials and manually-defined fiducials on the subject's MRI, and between the digitised head points and the canonical scalp mesh (excluding head-points below the nasion, given absence of the nose on the T1-weighted MRI). Lead-fields for each sensor were calculated for a dipole at each point in the

canonical cortical mesh, oriented normal to that mesh, and combined into a gain matrix, using the Nolte method implemented in FieldTrip (http://fieldtrip.fcdonders.nl/), based on fit to the inner skull mesh. Finally, these forward models were inverted using Multiple Sparse Priors (Friston et al., 2008), which were optimised by pooling over participants (Henson et al., 2011). This inversion was performed simultaneously for each condition of interest using a -500 ms to 500 ms time window of interest (time-locked to DP) and a 0 to 20 Hz frequency band of interest. The total energy within the post-DP time-window (i.e. 100 ms to 500 ms) and was then written to a 3D image in MNI space for each condition of interest and each participant.

Statistical Analyses

Statistics were first performed on the Global Field Power (RMS across all sensors) of the magnetometers and gradiometers, averaged over the pre-DP and the post-DP time-window of interest. Because the (rectified) GFP data were not normally distributed, we used the Wilcoxon Signed Ranks test to assess difference between conditions. Secondly, the averaged ERFs in each participant and condition were entered into a 3D (2D scalp topography x time) analysis using the cluster-based permutation test implemented in FieldTrip software (http://fieldtrip.fcdonders.nl/) to control for multiple comparisons over sensors and time points (Maris and Oostenveld, 2007). This analysis was performed separately for the magnetometers and RMS-gradiometers and implemented as follows. We used F-statistics to assess critical hypotheses. Contiguous sensor-time samples in which differences between conditions exceeded an uncorrected P-value < .05 were identified and grouped into clusters. For each cluster the sum of sampled F-values was computed and the cluster with the maximum sum was used as the cluster-level test statistic and compared to a randomized null distribution assuming no difference between conditions (obtained by 3000 random permutations of the conditions within subjects). The corrected p-value was estimated as the proportion of the randomization null distribution that exceeded the observed maximum cluster-level test statistic.

For source reconstruction analyses, 3-D images in source space for each of the 9 conditions of interest in each participant were entered in a factorial ANOVA implemented in SPM 8 (http://www.fil.ion.ucl.ac.uk/spm/) with a pooled error and correction for nonsphericity. We used the actual values derived from our neural segment prediction simulation to test for the predicted response profile (Source Day 1 = 0.45; Novel Day 1 = 0.55; Baseline Day 1 = 2; Source Day 2 = 0; Novel Day 2 = 2; Baseline Day 2 = 2; see Supplemental Information, section C and Figure S6). These values were mean-centred so that this contrast summed to zero. These maps were thresholded at $P < .001$ uncorrected and a correction for multiple comparisons made using Random Field Theory (Worsley et al., 1996). In an additional analysis, we restricted the search volume to areas identified as being activated in the pseudoword > word contrast for Untrained items (i.e. *Lexicality effect;* see Figure S2). P values were corrected for multiple comparisons within this search volume using Random Field Theory. This was confirmed by non-parametric statistical mapping of the same contrast which showed similar peaks of activity in left [x = -52 y = -22 z = 12 and x = -42 y = -32 z = 6] and right [x = 58 y = -16 z = 14] STG using a voxel height threshold whole-brain corrected.

Supplemental References

Davis, H., and Zerlin, S. (1966). Acoustic relations of the human vertex potential. J. Ac. Soc. Am. *39*, 109-116.

Ellis, B. W., Johns, M. W., Lancaster, R., Raptopoulos, P., Angelopoulos, N., and Priest, R. G. (1981). The St. Mary's Hospital sleep questionnaire: a study of reliability. Sleep *4*, 93-97.

Friston, K., Harrison, L., Daunizeau, J., Kiebel, S., Phillips, C., Trujillo-Barreto, N., Henson, R., et al. (2008). Multiple sparse priors for the M/EEG inverse problem. NeuroImage *39*, 1104-1120.Garagnani, M., Wennekers, T., Pulvermuller, F. (2008) A neuroanatomically grounded Hebbian-learning model of attention–language interactions in the human brain, European Journal of Neuroscience, 27(2), 492-513.

Garagnani, M., Pulvermuller, F. (2011) From sounds to words: A neurocomputational model of adaptation, inhibition and memory processes in auditory change detection*,* NeuroImage 54, 170-181.

Garagnani, M., Shtyrov, Y., Pulvermuller, F. (2009) Effects of attention on what is known and what is not: MEG evidence for functionally discrete memory circuits, Frontiers in Human Neuroscience, 3(10), doi:10.3389/neuro.09.010.2009.

Henson, R.N., Mouchlianitis, E., and Friston, K.J. (2009). MEG and EEG data fusion: Simultaneous localisation of face-evoked responses. Neuroimage *47*, 581-589.

Henson, R. N., Wakeman, D. G., Litvak, V., and Friston, K. J. (2011). A Parametric Empirical Bayesian Framework for the EEG/MEG Inverse Problem: Generative Models for Multi-Subject and Multi-Modal Integration. Front. Hum. Neurosci. *5*, 76.

Maris, E., and Oostenveld, R. (2007). Nonparametric statistical testing of EEG- and MEG-data. J. Neurosci. Meth. *164*, 177-190.

Mattout, J., Henson, R. N., and Friston, K. J. (2007). Canonical source reconstruction for MEG. Comp. Int. Neurosci. *2007*, 67613.

Worsley, K.J., Marrett, S., Neelin, P., Vandal, A.C., Friston, K.J.and Evans, A.C. (1996) A Unified Statistical Approach for Determining Significant Signals in Images of Cerebral Activation. Human Brain Mapping, *4*, 58-73.

Litvak, V., and Friston, K. (2008). Electromagnetic source reconstruction for group studies. NeuroImage *42*, 1490-1498.
